# Benchtop NMR-Based Metabolomics: First Steps for Biomedical Application

**DOI:** 10.3390/metabo13050614

**Published:** 2023-04-29

**Authors:** Pilar Alonso-Moreno, Ignacio Rodriguez, Jose Luis Izquierdo-Garcia

**Affiliations:** 1 NMR and Imaging in Biomedicine Group, Instituto Pluridisciplinar, Universidad Complutense de Madrid, 28040 Madrid, Spain; malons46@ucm.es (P.A.-M.); ignrodri@ucm.es (I.R.); 2 Department of Chemistry in Pharmaceutical Sciences, Pharmacy School, Universidad Complutense de Madrid, 28040 Madrid, Spain; 3 CIBER de Enfermedades Respiratorias (CIBERES), Instituto de Salud Carlos III, 28029 Madrid, Spain

**Keywords:** nuclear magnetic resonance, metabolomics, benchtop, low field, diabetes, tuberculosis, diagnosis, biomarkers, metabolites

## Abstract

Nuclear magnetic resonance (NMR)-based metabolomics is a valuable tool for identifying biomarkers and understanding the underlying metabolic changes associated with various diseases. However, the translation of metabolomics analysis to clinical practice has been limited by the high cost and large size of traditional high-resolution NMR spectrometers. Benchtop NMR, a compact and low-cost alternative, offers the potential to overcome these limitations and facilitate the wider use of NMR-based metabolomics in clinical settings. This review summarizes the current state of benchtop NMR for clinical applications where benchtop NMR has demonstrated the ability to reproducibly detect changes in metabolite levels associated with diseases such as type 2 diabetes and tuberculosis. Benchtop NMR has been used to identify metabolic biomarkers in a range of biofluids, including urine, blood plasma and saliva. However, further research is needed to optimize the use of benchtop NMR for clinical applications and to identify additional biomarkers that can be used to monitor and manage a range of diseases. Overall, benchtop NMR has the potential to revolutionize the way metabolomics is used in clinical practice, providing a more accessible and cost-effective way to study metabolism and identify biomarkers for disease diagnosis, prognosis, and treatment.

## 1. Introduction

Healthcare surveillance, which is critical for personalized medicine, is typically based on the observation of symptoms or the periodic evaluation of health-related biomarkers by a general practitioner. The first step in identifying health conditions throughout the body is usually the analysis of body fluid samples, which often only contain a small number of specific molecules that can distinguish between healthy and diseased states. However, basic and comprehensive metabolic and lipid panels used to assess health status do not have sufficient specificity to identify the early signs of a specific disease. The metabolome, on the other hand, reflects early and specific changes in the pathophysiological state of biological systems. Because small changes in enzyme concentrations or activities can lead to large changes in metabolite levels, the metabolome is considered the amplified output of a biological system. Metabolomic data is currently mainly generated through nuclear magnetic resonance (NMR) spectroscopy and mass spectrometry (MS). While MS has higher sensitivity, NMR spectroscopy is more robust and reproducible [1], making it a preferred tool for analyzing clinical samples. However, the translation of NMR-based metabolomics to a healthcare setting has been limited mainly by two factors: cost and portability. Cost: High-resolution (HR)-NMR spectroscopy instruments can be expensive to purchase. In addition, while the maintenance and operation cost of these instruments is generally lower than other metabolomic techniques such as MS, the cost of liquid helium required to operate the instrument can be a barrier for some clinical sites due to its price. Portability: HR-NMR spectroscopy instruments are typically large, heavy and require specialized facilities for installation and operation.

Benchtop NMR instruments retain many of the qualities of their parent techniques, such as being automatable and fast, allowing for higher sample throughput and greater scalability, but they have also addressed some of the previously noted limitations. Specifically, benchtop NMR instruments are generally more affordable and accessible than high-resolution NMR, which can facilitate broader adoption of NMR-based metabolomics in clinical research. Additionally, they are more portable and can be used in a variety of settings, including clinical and field-based environments. Benchtop-NMR technology uses permanent magnets, which have a lower magnetic field than the more expensive cryogen-cooled superconducting NMR spectrometers. Until recently, the magnetic field homogeneity of benchtop NMR spectrometers was not sufficient to record spectra with sufficient resolution. However, recent advances have resulted in the development of benchtop cryogen-free magnets operating at either 60, 80, or 90 MHz that overcome this issue [2]. The main advantages of these instruments include their affordable purchase price, low maintenance cost due to the absence of cryogenic fluids, and small size. Benchtop NMR spectrometers have been used for educational [3] or industrial applications [4,5], and there are no apparent limitations to transferring HR-NMR metabolomic results to a benchtop NMR for clinical use [6,7]. This review aims to introduce the potential of using benchtop NMR metabolomics for clinical applications.

## 2. Benchtop NMR Technical Developments

### 2.1. Benchtop NMR Spectrometers

There are currently six brands of benchtop NMR instruments available on the market: Bruker, Magritek, Nanalysis, Oxford Instruments, ThermoFisher, and Q-Magnetics. These instruments have magnetic field strengths ranging from 43 MHz to 125 MHz (^1^H frequency), and spectrometer weights ranging from 5 kg to 172 kg. Table 1 presents the specifications of the available instruments based on information from the manufacturers’ websites and brochures. It is worth noting that prices, which are not listed in Table 1, may also be a factor to consider when choosing an instrument. In addition to the benchtop NMR instruments listed, there are also lower field systems that are not suitable for metabolomics, such as the Oxford Instruments MQC+ and MQR, which have field strengths ranging from 5 MHz to 23 MHz (^1^H frequency) and use tubes ranging from 5 mm to 60 mm. When deciding which instrument to purchase, buyers should consider factors such as the presence of an autosampler, the type of lock, the ability to suppress the solvent signal, and processing software, in addition to the magnetic field strength. There is no one “best” instrument, so buyers should choose the one that best meets their needs.

### 2.2. One-Dimensional Benchtop NMR Spectroscopy

Benchtop NMR spectrometers are capable of detecting several nuclei, depending on the hardware and equipment probes [6]. While some nuclei are commonly studied in benchtop NMR, such as ^1^H, ^13^C, ^19^F, and ^31^P, there are other nuclei that have high natural abundance and a suitable gyromagnetic ratio for use in benchtop NMR, such as ^7^Li, ^11^B, ^13^Na, and ^27^Al [18].

**Figure 1:**
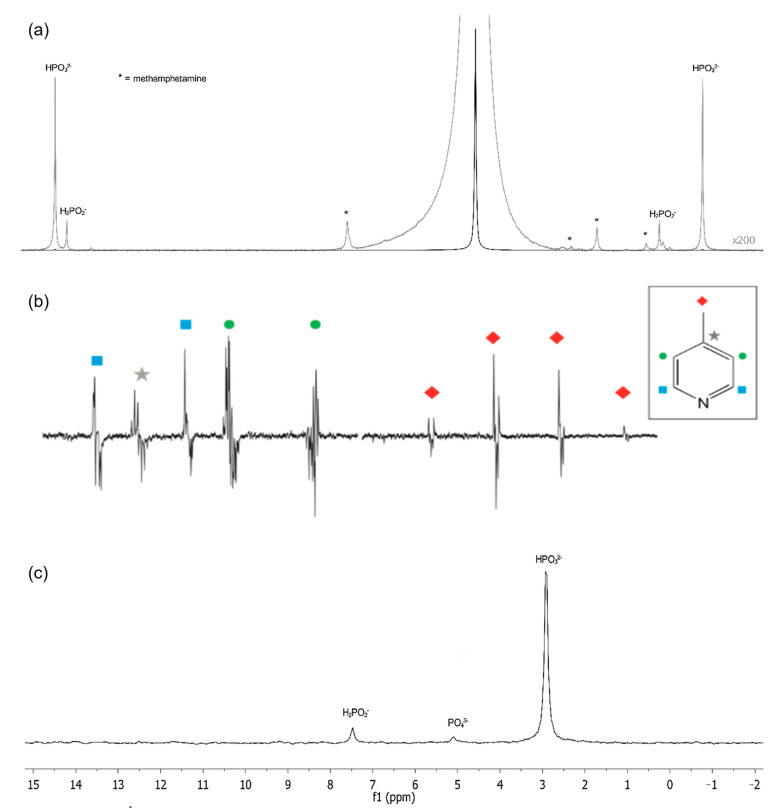
1D NMR spectra from different isotopes. (**a**) ^1^H (200× magnification) and (**c**) ^31^P NMR of Caustic Waste from Toluene Extraction of methamphetamine reaction. (**b**) 1D SABRE-hyperpolarized ^13^C NMR spectra of 4-methylpyridine with active SABRE catalyst and acquired following a 90°. NMR assignments from each non-equivalent carbon signals are highlighted by color and shape. Each carbon from the molecule has been assigned to Reproduced with permission from B. Bogun et al. [23] (**a**) &(**c**); and A. D. Robinson et al. [24] (**b**).

In benchtop NMR, ^1^H is the most commonly studied nucleus due to its high sensitivity and abundant nature in organic molecules [6,19]. ^1^H signal is receptive and easily observed, but the narrow spectral window can lead to a poor shift dispersion [18,20] (Figure 1a). ^19^F nucleus is also particularly popular due to its sensitivity and wide frequency range, which is similar to that of ^1^H [6,19,21]. ^19^F has a high gyromagnetic ratio and a 100% natural abundance, making it an interesting nucleus for modern pharmaceutical products, reaction monitoring, drug identification, and organic compound analysis [18,22].

Secondary probes can be used to detect complementary nuclei, such as ^13^C or ^31^P, but there may be limitations in the medium-field. The element ^13^C is important for organic molecules but can only be detected in high concentrations at this intensity due to its low natural abundance and modest gyromagnetic ratio [6,22]. Nevertheless, ^13^C provides good resolution and easy interpretation due to its narrow spectral window (0–250 ppm) and is useful for structural determinations. It is typically analyzed with ^13^C 1D NMR, ^13^C-DEPT, and ^1^H-^13^C 2D NMR [22]. While ^13^C has low limit of detection (LOD) values, its sensitivity can be increased through techniques such as hyperpolarization, combined with optimal technology [21]. Some groups have investigated hyperpolarization methods of ^13^C to improve signal resolution and decrease significant peak overlap [22,24], including the use of a parahydrogen-based hyperpolarization technique known as signal amplification by reversible exchange (SABRE) (Figure 1b). This approach has demonstrated high sensitivity in 1D and 2D NMR with easy peak interpretation that includes peaks with frequency lower than 0.5 ppm [24,25,26]. The element ^31^P is less sensitive than ^19^F and ^1^H but has a 100% natural abundance, and has been used in food analysis, pharmaceutical products analysis, inorganic applications, biochemistry, and in compounds such as organophosphines, inorganic phosphates, phospholipids, and catalysis organometallic reactions [18,22,23,27] (Figure 1c).

Finally, other nuclei such as ^7^Li in the mining industry [28], ^129^Xe in biosensors [29], and ^207^Pb in various applications [30], are special to specific areas and may not be as useful for biomedical applications.

### 2.3. Two-Dimensional Benchtop NMR Spectroscopy

Two-dimensional (2D) NMR is a powerful technique for improving peak resolution and identifying unknown metabolites [31,32,33]. This technique separates signals based on their chemical shifts and J-coupling values, providing both structural and quantitative information, making it particularly useful for complex biochemical and chemical mixtures [33,34,35]. However, the long preparation and acquisition times make it a laborious process. To overcome this limitation, researchers are exploring new computational and machine-learning methods [31,32,33]. Moreover, due to the high time and resource requirements, only a few samples are usually studied with this technique [31,32,34], and researchers have developed faster versions of 2D NMR to improve throughput [31,34].

**Figure 2:**
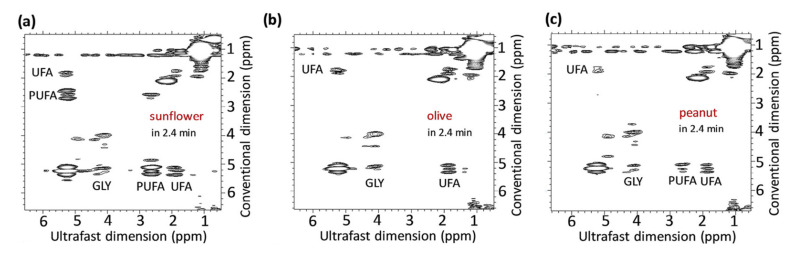
2D UF COSY spectra of sunflower (**a**), olive (**b**), and peanut (**c**) oil samples at 43 MHz. Assignments: relative concentration of glycerol (GLY), polyunsaturated fatty acids (PUFA) and unsaturated fatty acids (UFA). Reproduced with permission from B. Gouilleux et al. [31].

The faster versions of 2D NMR are described in many articles, with some based on repetition time reduction, whereas others use selective pulses [34]. Among these methods, ultrafast or single-scan NMR has emerged as the most useful, acquiring a complete experiment in a single scan by exploiting spatial parallelization processes [31,34,36]. However, faster processes can cause a decrease in resolution or a more complex calibration [33]. The ultrafast 2D NMR method has shown its usefulness in two different fields, particularly in the oil industry, where researchers have explored this technique for screening and discriminating between different oils [4]. Gouilleux et al. [31] were the first to implement ultrafast 2D NMR on a benchtop spectrometer. They used a Magritek Spinsolve Benchtop spectrometer operating at a frequency of 43.62 MHz and conducted a COSY (Correlated Spectroscopy) experiment in a gradient coil to advance solvent suppression methods. The team concluded that the equipment offered excellent analytical performance and screening speed (Figure 2). In the same vein, Gouilleux et al. evaluated the potential of several benchtop instruments for screening various vegetable oils, including olive, hazelnut, sesame, rapeseed, corn, and sunflower, using ultrafast 2D NMR and detecting the adulteration of olive oil [36]. They used a Magritek Spinsolve spectrometer at a frequency of 43.62 MHz and found that the equipment proposed a benefit in discriminating between the different oils, but its performance could be increased with stronger magnetic field strengths and homogeneity.

**Figure 3:**
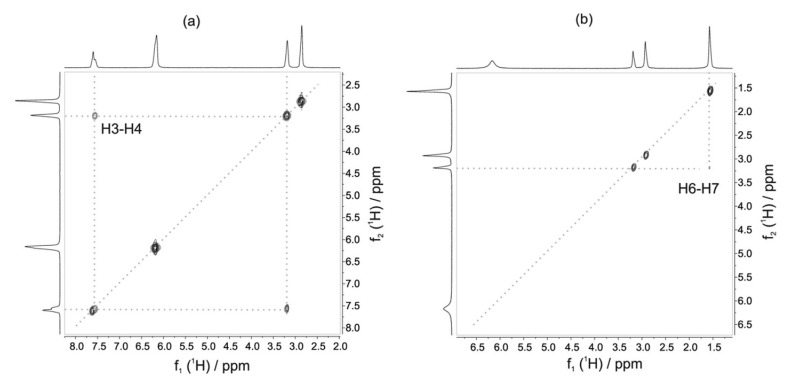
2D ^1^H-^1^H COSY spectrum of the formation of methyl formate (**a**) and methyl acetate (**b**). Reproduced with permission from A. Friebel et al. [39].

In addition, benchtop NMR has shown potential in studying kinetic reactions for identifying and quantifying their products [37]. For instance, Koos et al. investigated the chemical distinction and abundance of enantiomers and the formation of diastereomers using a Magritek/ACT prototype spectrometer at a frequency of 42.8 MHz [38]. Their study demonstrated that the equipment provided high chemical resolution and the possibility to differentiate small molecules. Similarly, Friebel et al. [39] probed a new stationary flow reactor setup to monitor the composition of the reaction mixture and identify unknown products. They tested the transformation and kinetics of methyl formate from methanol and formic acid, and the reaction of methanol and acetyl acid to produce methyl acetate (Figure 3). Using a Magritek Spinsolve Carbon NMR spectrometer at a frequency of 42.5 MHz, they analyzed 1D 1H-NMR and 2D with ^1^H-^1^H COSY, Heteronuclear Chemical Shift Correlation (HETCOR) [40], and Heteronuclear Multiple Bonding Correlation (HMBC) [41]. Their findings indicated similar results to those reported in the literature and suggest the potential extension of benchtop NMR to reaction monitoring.

Furthermore, benchtop NMR has been explored for monitorization of hydrogenation reactions. Gołowicz et al. [42] studied the possibility of increasing resolution and sensitivity by combining parahydrogen-induced polarization and fast correlation spectroscopy. They used a Magritek Carbon spectrometer at a frequency of 43 MHz and analyzed the samples using one-dimensional ^1^H and 2D with a 2D double-quantum filtered correlation spectroscopy (NUS DQF-COSY). Their results showed that benchtop NMR may be used to monitor hydrogenation reactions.

Several authors explored different methods to improve material research. Leutzsch et al. [43] used benchtop equipment to study heterogeneous catalytic reactions with the goal of understanding the interactions between adsorbates and adsorbents, specifically in terms of the access of 2-butanone to surface sites. They used a Bruker AV spectrometer (85 MHz for 1H) to obtain information about porous heterogeneous catalyst materials. They concluded that this method can provide more information about the behavior of liquids inside catalysts and may predict catalyst performance [43,44].

**Figure 4:**
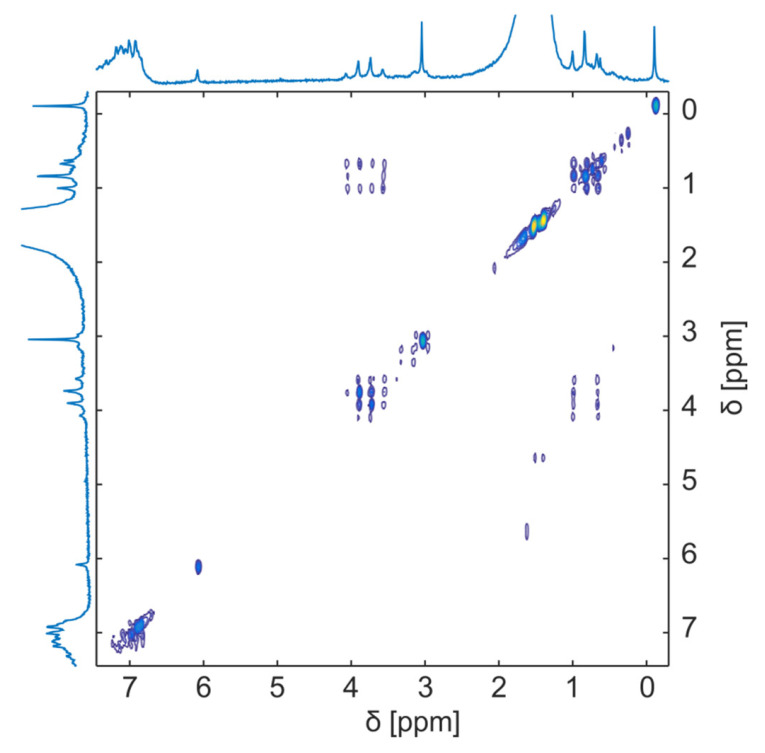
2D ^1^H-^1^H DOSY spectrum of deprotonation and diacylation reaction from α-fluoro-β-keto esters to α- fluoro-α,β-unsaturated esters at room temperature after 22 h. Reproduced with permission from D.Weidener et al. [45].

Weidener et al. [45] focused on the need to understand pharmacological and industrial reactions. They monitored the deprotonation and diacylation from α-fluoro-β-keto esters to α- fluoro-α,β-unsaturated esters using a Magritek SpinSolve 1 T NMR spectrometer. They conducted a one-dimensional ^1^H (42.5 MHz) and ^19^F (40.1 MHz) and two-dimensional ^1^H-^1^H COSY. They found that this equipment provided information about the mechanism and kinetics of the reaction, which is essential for optimizing synthetic routes (Figure 4). In a different study, Steimers et al. [33] compared 2D non-uniform sampling (NUS), a non-linear method in which some fractions of data are skipped but are usually measured, with 2D NUS-heteronuclear single quantum coherence (HSQC) and a 1D ^1^H-NMR spectral. They selected the glycerol reaction that produces five products with similar chemistry and used the Magritek Spinsolve Carbon NMR Spectrometer at a frequency of 42.5 MHz. They obtained similar results with both approaches and concluded that 2D NUS is a method to improve spectral resolution.

Two-dimensional benchtop NMR spectroscopy has also been applied to environmental studies [46,47]. For instance, Fallaise et al. [47] investigated the water located under soil, which is an important source of groundwater, but is often affected by nearby industrial activities. They characterized the water compound in two different situations: one near industrial activities and the other without industrial activities. Using an Oxford Instruments Pulsar benchtop NMR spectrometer at a frequency of 60 MHz, they obtained one- and two-dimensional NMR ^1^H J-resolved spectroscopy (JRES) [48,49]). Their conclusion was that this equipment could be used to identify different contaminants and remediation strategies [47].

Apart from these studies, very few have employed heteronuclear 2D techniques other than ^1^H-^1^H. For example, ^1^H-^13^C with HETCOR was used to demonstrate the SABRE efficiency in two-dimensional acquisition [24], and ^1^H-^19^F with Heteronuclear Overhauser Spectroscopy (HOESY) was used to determine the internuclear distance and conformational dynamics of fluorinated pharmaceutical compounds [50].

### 2.4. New Pulse Sequences

The limitations of benchtop NMR spectrometers compared to high-field NMR instruments are well known, including lower sensitivity, reduced spectral resolution, and greater spectral complexity due to overlapping resonances. These limitations pose challenges for performing certain types of experiments and extracting specific information from the spectra. One of the main limitations is the peak overlaps of the different compounds being targeted. Therefore, the development of new pulse sequences tailored to benchtop NMR is necessary to enhance the capabilities of this technology and expand its range of applications. In this regard, coherence selection, encoding chemical shift, and diffusion information are promising areas for the development of new pulse sequences [51].

Efficient solvent elimination schemes are essential to improve the detection of relevant analytes, and several such schemes have been explored at high magnetic fields. The main criteria to define their efficiency include decreasing the intensity of the solvent signal(s), selectivity of solvent suppression, and analytical features such as repeatability and robustness [52]. However, benchtop NMR has its own specificities with regards to solvent suppression, making selectivity issues the main difficulty in implementing solvent suppression schemes [51]. While the simplest and most popular solvent suppression scheme at high field is the continuous wave presaturation (Sat) [53], it is not the most robust scheme on benchtop spectrometers. Two very different approaches to removing unwanted solvent peaks, both relying on a clever use of gradient pulses, are WET-180-NOESY and WATERGATE W5 [54]. WET-180-NOESY selectively defocuses the solvent magnetization while leaving those of relevant analytes unaffected before excitation. On the other hand, WATERGATE W5 selectively refocuses all magnetizations except those from user-defined regions that contain the solvent peaks. Therefore, the development of specific sequences for solvent suppression on benchtop NMR is crucial for improving the detection of relevant analytes.

Spatial encoding techniques, which were originally developed for MRI, have been adapted for use in high field NMR [55]. These techniques have recently been introduced to benchtop spectrometers in the form of ultrafast multi-dimensional NMR, which uses a helicoidal magnetization winding created by linearly frequency-swept pulses with gradients in place of the conventional time-incremented period. During the acquisition process, this information is decoded through an echo-planar spectroscopic imaging (EPSI) scheme, allowing for the recording of a complete 2D matrix in a single scan. This pulse sequence has the same structure as conventional multidimensional experiments, but it offers faster acquisition times and improved spatial resolution [56].

Spectral editing techniques have been developed to address the limitations of benchtop NMR spectroscopy, such as limited spectral dispersion leading to signal superpositions and difficulties in compound identification and quantification. Two popular techniques for aiding in identification and quantification are pure shift NMR [57,58] and diffusion-ordered spectroscopy (DOSY) [59,60,61]. Pure shift NMR, also known as “chemical shift spectra” or “homonuclear broadband decoupling,” disentangles overlapped proton NMR spectra, with methods such as the 1D projection of 45° tilted 2D J-resolved experiment [62] and the Zangger-Sterk (ZS) method, based on a low intensity gradient combined with a selective shaped pulse to spatially encode the signal frequency into the tube [63]. DOSY separates spectra based on the molecular weight of the associated molecule. This is possible due to the relationship between molecular weight and diffusion coefficient. However, these techniques suffer from either poor sensitivity (pure shift NMR) or the need for specialized equipment (DOSY). An alternative approach is subspectral editing using optimal control pulses, where selective radiofrequency pulses are designed using optimal control theory, as demonstrated in recent research [64].

To achieve even more powerful analytical capabilities, it may be beneficial to combine several of the existing techniques. A particularly promising direction is to integrate solvent suppression schemes with spatially encoded methods. For example, by combining the spatial encoding approach with pure-shift NMR spectroscopy, a variety of 2D pure-shift experiments could be designed, such as TOCSY-PSYCHE [65] or pure-shift HSQC based on a BIRD filter [66], which would offer greater versatility and more effective identification and quantification of individual compounds. Such developments could bring benchtop NMR spectroscopy to the forefront of advanced analytical tools, with a broad range of applications in diverse fields such as pharmaceuticals, clinical diagnostics, and environmental analysis.

## 3. Benchtop NMR-Based Metabolomics

### 3.1. Tuberculosis Research

Tuberculosis (TB) is the leading cause of death from infectious diseases worldwide. It most commonly affects the lungs, but can also affect other parts of the body, such as the brain, kidneys, and bones. TB is transmitted through the air when an infected person speaks, coughs, or sneezes, and is inhaled by others. Misdiagnosis and late detection of the disease increases the risk of transmission and infection of Mycobacterium tuberculosis. While culture is currently the gold standard for diagnosing TB, it takes approximately 4-6 weeks to obtain results [67]. HR-NMR spectroscopy is a commonly used metabolomics technique in various biological samples for TB biomarker identification, improving diagnosis and treatment monitoring [68,69]. For example, a study published in the Journal of Proteome Research [70] identified several metabolites in the blood serum of TB patients that were differentially expressed compared to healthy controls, including 1-methylhistidine, acetoacetate, acetone, glutamate, glutamine, isoleucine, lactate, lysine, nicotinate, phenylalanine, pyruvate, tyrosine, alanine, formate, glycine, glycerolphosphocholine, and low-density lipoproteins. Another study published in Infection, Genetics and Evolution [71] used HR-NMR to identify metabolites in cerebrospinal fluid samples that were able to differentiate TB meningitis from viral meningitis, bacterial meningitis, and meningitis-negative groups. However, as we mentioned earlier, HR-NMR spectroscopy has several limitations for clinical use. In an effort to find alternative clinical approaches, our group at Complutense University has explored the use of benchtop NMR spectrometry as a metabolomic tool for TB diagnosis. We hypothesized that identifying a metabolic pattern in urine or blood plasma using NMR technology could provide a new approach for TB diagnosis. To test this hypothesis, three independent studies were conducted.

**Figure 5:**
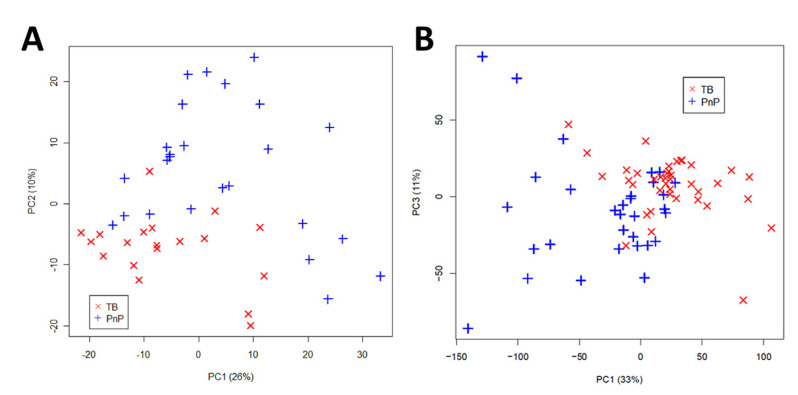
Principal Component Analysis (PCA) score plots of urine spectra analyzed by (**A**) HR-NMR of untreated TB patients (*n* = 19) and pneumococcal pneumonia patients (*n* = 25); (**B**) benchtop NMR of untreated TB patients (*n* = 39) and pneumococcal pneumonia patients (*n* = 31). TB, tuberculosis; PnP, pneumococcal pneumonia; PC, principal component. Reproduced with permission from J.L. Izquierdo-Garcia et al. [72].

TB diagnosis in adults: The objective of this study [72] was to identify a metabolic profile in TB patients using HR-NMR spectroscopy and to validate this characteristic metabolic profile using benchtop NMR spectroscopy. To achieve this, urine samples from TB patients (*n* = 19) and pneumococcal pneumonia (PnP) patients (*n* = 25) were analyzed using a high-resolution Bruker Advance spectrometer operating at 700 MHz. The urine spectra provided very good discrimination between the two groups based on unsupervised principal component analysis (PCA) (Figure 5A). Seven metabolic biomarkers were identified that significantly differed between the groups. A modified partial least squares (PLS) algorithm was developed as a predictive model with a classification accuracy of 100% for test samples.

**Figure 6:**
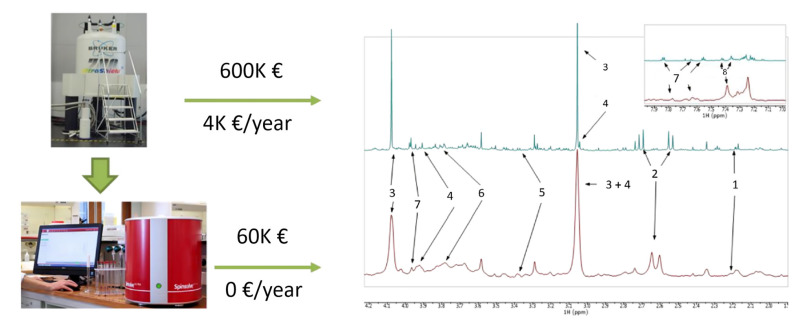
^1^H NMR spectra of urine samples from tuberculosis patients acquired by (top) 700 MHz Bruker NMR spectrometer, and (bottom) 80 MHz Magritek NMR spectrometer. Approximate purchase and maintenance costs of HR-NMR and benchtop NMR spectrometers are shown. Assignments: 1, 2-aminoadipic acid; 2, citrate; 3, creatinine; 4, creatine; 5, glucose; 6, mannitol; 7, hippurate; 8, phenylalanine. Reproduced with permission from J.L. Izquierdo-Garcia et al. [72].

The transition to a benchtop NMR-based metabolic approach was carried out using a Magritek Spinsolve 60 Ultra Benchtop spectrometer (Magritek GmbH, Aachen, Germany) at a frequency of 60 MHz. Urine samples were analyzed using a 1D PRESAT pulse sequence and averaged over 64 acquisitions. While benchtop NMR spectra had lower resolution than HR-NMR spectra, the seven metabolites identified using HR-NMR spectroscopy were also identified and quantified in benchtop NMR spectra (Figure 6). The good correlation between high-resolution and benchtop NMR metabolic signals suggests that affordable technology could be used for clinical applications, both in terms of spectrometer purchase and maintenance costs. As proof of concept, urine samples from TB patients (*n* = 39) and PnP patients (*n* = 31) were analyzed using a Magritek 60MHz Spinsolve spectrometer. Although the discrimination between the two groups provided by PCA analysis was good, it was not as effective as the discrimination obtained with a HR-NMR-based metabolomic approach (Figure 5B). PLS-DA provided a classification accuracy of 100% by test samples.

**Figure 7:**
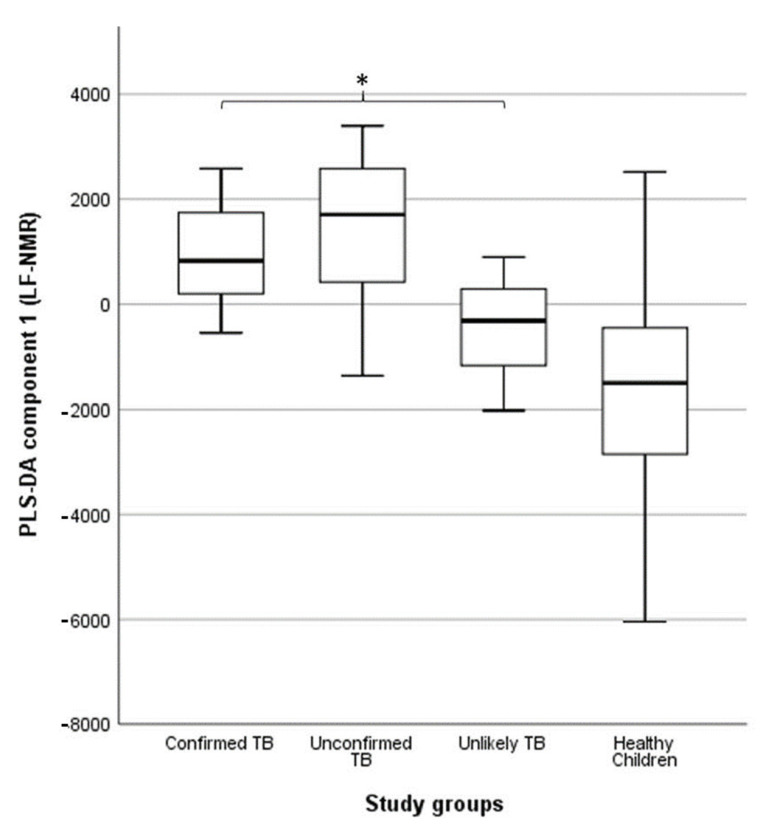
Association between partial least squares discriminant analysis (PLS-DA) scores and study groups in 109 urine spectra acquired by benchtop proton (^1^H) nuclear magnetic resonance. The central horizontal line within the boxes represents the median. The boxes comprise the first and third quartiles, the tiles indicate the maximum and minimum values, and the asterisk indicates statistically significant differences (*p*-value < 0.05) between groups. TB: tuberculosis. Reproduced with permission from P. Comella-del-Barrio et al. [73].

TB diagnosis in children: under-detection of childhood TB is common in low- and middle-income countries because its clinical presentation overlaps with other respiratory infections. Children have low sputum bacillary loads and are often unable to produce sputum, making it difficult to diagnose TB in this population. We examined whether urine NMR-based metabolomics could be used to identify differences in the metabolic response of children with different levels of diagnostic certainty for TB [73]. We compared the metabolic patterns of 62 children with signs and symptoms of TB (six children with bacteriologically confirmed TB, 52 children with unconfirmed TB, and four children with unlikely TB) to 55 apparently healthy children. Urine metabolic fingerprints were identified using a Magritek 60 MHz Spinsolve spectrometer. We observed differences in the metabolic fingerprint of children with bacteriologically confirmed and unconfirmed TB compared to those with unlikely TB (*p* = 0.041 and *p* = 0.013, respectively) (Figure 7). These differences in the metabolic fingerprint in children with different levels of diagnostic certainty for TB could contribute to a more accurate characterization of TB in the pediatric population.

**Figure 8:**
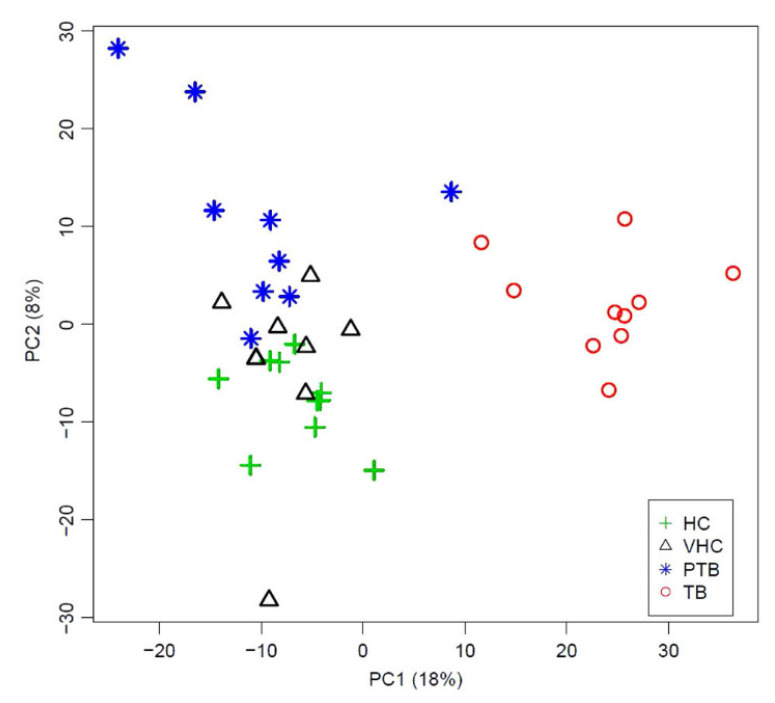
Principal Component Analysis (PCA) of plasma spectra measured using benchtop proton (^1^H) nuclear magnetic resonance from cows diagnosed with bovine tuberculosis (*n* = 10), cows diagnosed with paratuberculosis (*n* = 10), paratuberculosis-vaccinated healthy controls (*n* = 10) and healthy paratuberculosis-unvaccinated controls (*n* = 10). PC1-PC2 score plot shows clear discrimination between tuberculosis samples and paratuberculosis, vaccinated healthy control and unvaccinated healthy control samples across PC1, whereas healthy unvaccinated control and paratuberculosis samples are separated across PC2. HC = uninfected/paratuberculosis-unvaccinated healthy control. VHC = uninfected/paratuberculosis-vaccinated healthy control. PTB = paratuberculosis. TB = tuberculosis. PC = principal component. Reproduced with permission from J. Ruiz-Cabello et al. [74].

Bovine TB diagnosis: despite significant efforts and control strategies, bovine tuberculosis (BTB) continues to be a major source of health and socio-economic concern. The standard method used in BTB eradication programs for in vivo detection is the tuberculin skin test. However, the specificity of the tuberculin skin test is affected by infection with non-tuberculous mycobacteria (e.g., Paratuberculosis, PTB) or by vaccination, leading to incorrect diagnosis in some animals. We aimed to identify a plasma metabolic BTB profile using HR-NMR spectroscopy and to measure this characteristic BTB metabolic profile using benchtop NMR as an affordable molecular technology for BTB diagnosis [74]. We identified 14 metabolites that were significantly different between BTB and control animals. Plasma fingerprinting using a Magritek 60 MHz Spinsolve spectrometer was able to differentiate TB subjects from uninfected animals, as well as PTB and PTB-vaccinated subjects who may provide a BTB-false positive (Figure 8), highlighting the potential use of benchtop NMR-based metabolomics as a complementary or alternative diagnostic tool to current methods.

### 3.2. Diabetes Research

Type 2 diabetes is a metabolic disease that affects millions of people worldwide. According to the World Health Organization (WHO), the number of cases is expected to increase by 122% by 2025 due to demographic, cultural, and aging factors in developing countries [75,76]. The majority of cases of type 2 diabetes are characterized by a deficiency in insulin synthesis, secretion, or action, which is caused by the inability of pancreatic beta cells to produce enough insulin [77]. Uncontrolled diabetes can lead to cardiovascular, renal, and ocular diseases and disorders as primary complications, but it can also result in other issues such as neuropathy, amputations, and retinopathy [75]. Symptoms of type 2 diabetes include increased thirst, increased urination, and unexplained weight loss [78], but many patients are not diagnosed early on due to the absence of symptoms or the presence of non-specific ones [75,79]. NMR-based metabolomics can be useful for researching and detecting type 2 diabetes early on [80,81]. However, HR-NMR has limitations as explained before, thus some researchers have turned to benchtop NMR spectroscopy as a more practical method for use in clinical settings [6,7].

**Figure 9:**
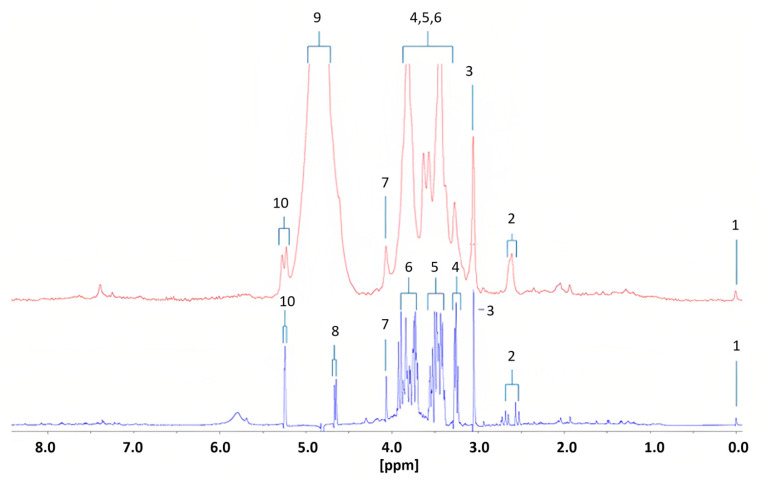
Diabetic urinary ^1^H NMR profile acquired with 60 (red) and 400 MHz (blue) NMR spectrometers. Assignments; ts: 1, TSP-Si(CH3)3; 2, Citrate-A/B-CH2CO2−; 3, Cn/Creatine > N-CH_3_; 4, β-Glucose-C2-H; 5, α- and β-Glucose-C4-H/C5-H, and α-Glucose-C2-H; 6, α- and β-Glucose-C3-H/C5-H/C6-H2; 7, Cn-CH_2_; 8, β-Glucose-C1-H; 9, H2O/HOD-OH; 10, α-Glucose-C1-H. Reproduced with permission from B.C. Percival et al. [21].

Percival et al. [21] conducted the first study using benchtop NMR spectroscopy for diabetes research. They identified 15 metabolic biomarkers that could distinguish diabetic patients from healthy controls using a 60 MHz Magritek Spinsolve Ultra NMR spectrometer and compared the results with those obtained from a HR Bruker 400 MHz Avance-1 NMR spectrometer. The metabolomic profiles were determined in urine samples from 10 diabetic patients and 14 healthy subjects. The differences between the two frequencies were significant, with an expansion of multiplet signals observed at lower frequency (Figure 9). Of the 15 potential biomarkers identified, seven were upregulated in type 2 diabetic individuals, including alpha-glucose and acetone, which are commonly observed in diabetes. The remaining biomarkers were downregulated in the benchtop NMR (Table 2). However, the study had several limitations. Firstly, the benchtop systems were not suitable for observing polyuria, a condition characterized by excessively high urine volume in patients with uncontrolled or poorly controlled type 2 diabetes. This is due to significant resonance overlap problems for creatine, which is a key biomarker for polyuria. Secondly, the precision and computational models of the study needed improvement by incorporating predictor metabolic variables and eliminating datasets before removing secondary disturbances in the analysis. Finally, the low intensity of some benchtop systems could cause presaturation of H2O/HOD signals, which could be observed in resonances close to short-range and long-range coupling effects.

**Figure 10:**
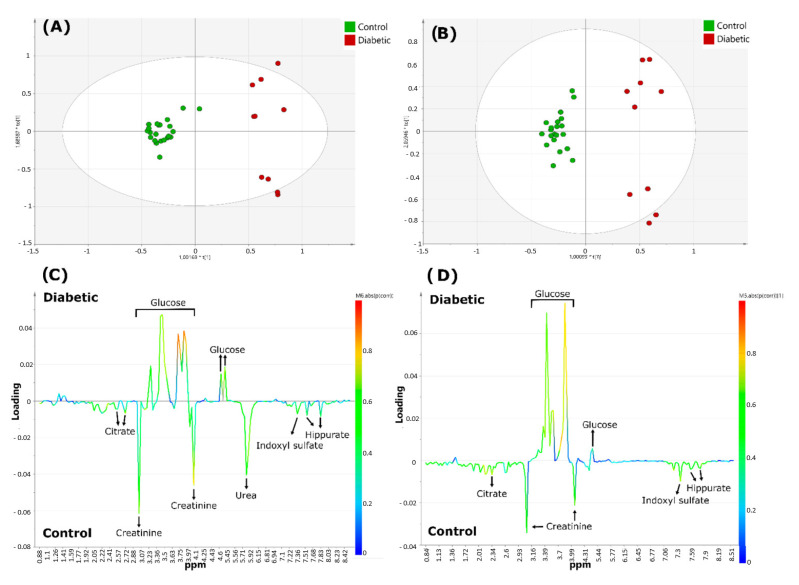
Metabolomics analysis of diabetics ^1^H NMR urinary profiles versus healthy controls acquired with 60 and 400 MHz NMR spectrometers. Scores plots (**A**,**B**), and corresponding loading-plots (**C**,**D**) from orthogonal partial-least squares discriminant analysis (OPLS-DA), data obtained using 400 (**A**,**C**) or 60 MHz (**B**,**D**) NMR spectrometers. The color of the signals in (**C**,**D**) correspond to the metabolites contributing most greatly towards the separation between diabetics and healthy control group samples. The color bar next to the right-hand side of these plots indicates the level of importance of the metabolites discriminating between classes and the colors represented more (red) or least (blue) significant metabolites. Reproduced with permission from J. Leenders et al. [82].

The same research group continued to validate the use of benchtop NMR with a second study that improved spectral resolution through the acquisition of a two-dimensional (2D) NMR profile at low frequency (LF) [82]. They used the same samples as in their previous study [15] to reproduce the previous results using ^1^H-^1^H 2D acquisitions correlation spectroscopy (COSY) at 60 MHz. Urea was the only metabolite identified with HR-NMR that was not detected with the benchtop NMR spectrometer (Figure 10). This could have been due to the presence of unassigned doublet resonances detectable at the low field frequency or because these signals were located near the residual water signal and could be associated with solvent suppression sequences used. Therefore, the authors proposed further research to address this limitation. Additionally, the small number of participants in this study made it difficult to draw broad conclusions. However, they concluded that benchtop systems could successfully reproduce metabolomic results similar to those obtained with high-frequency systems.

**Figure 11:**
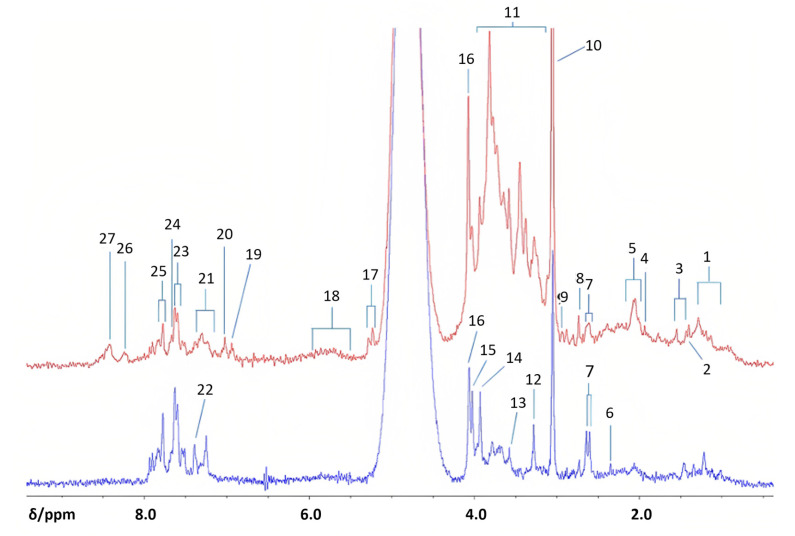
Benchtop ^1^H NMR analysis of type 2 diabetics and health controls urine samples. Profiles of diabetics (top, red) and healthy controls (bottom, blue) were acquired with a 60 MHz NMR spectrometer. Abbreviations: 1, branched-chain amino acid-CH3 functions (i.e., those of isoleucine, leucine and valine); 2, 3-D-hydroxybutyrate-CH3; 3, lactate-CH3; 4, acetate-CH3; 5, –NHCOCH3 functions of N-acetylsugars (free or present as side-chain residues in urinary N-acetylated glycoproteins) and/or N-acetylamino acids, e.g., N-acetylaspartate; 6, pyruvate-CH3; 7, citrate-CH2CO2; 8, dimethylamine-N(CH3)2; 9, dimethylglycine-N(CH3)2; 10, creatine/creatinine>N-CH3; 11, bulk-and-glucose C2H-C6H ring protons; 12, trimethylamine-N-oxide ON(CH3)3; 13, glycine-CH2; 14, guanidoacetate-NH-CH2-CO2; 15, creatine-CH2/hippurate-NH-CH2-CO2; 16, creatinine-NH-CH2-CO2; 17, -glucose-C1H; 18, urea-CO-NH2; 19, 3-(3-hydroxyphenyl)-3- hydroxypropanoate-C2H, C4H, C6H; 20, phenol-C2H, C4H, C6H; 21, indoxyl sulphate-C3H, C4H; 22, indoxyl sulphate = CH-NH−; 23, indoxyl sulphate-C2H/hippurate-C3H, C5H; 24, hippurate-C4H; 25, hippurate-C2H, C6H; 26, unassigned pyrimidine resonance, possibly hypoxanthine-CHs; 27, formate H-CO2. Reproduced with permission from M. Edgar et al. [79].

Edgar et al. sought to confirm the practical application of benchtop NMR in comparison to HR-NMR [79]. In this study, urine samples from 10 diabetic patients and 14 healthy subjects were analyzed using a 60 MHz Magritek Spinsolve Benchtop NMR spectrometer and three HR-NMR spectrometers: a Bruker 400 MHz Avance-1 NMR spectrometer (Coventry, UK), a JEOL ECS-400 spectrometer (Tokyo, Japan), and a Bruker 700 MHz AV-III NMR spectrometer equipped with a ^1^H (^13^C/^15^N) TCI cryoprobe (Coventry, UK). While the results obtained at 400 and 700 MHz differed in the number of quantifiable resonances, the benchtop spectrometer was able to demonstrate a significant upregulation of citrate and creatine in diabetic urine samples (Figure 11). The researchers analyzed 15 potential metabolites, of which three were upregulated and four were downregulated with benchtop NMR (Table 2). They suggested that the exploration of classical and non-classical biomarkers could be enhanced and monitored for diabetes and kidney complications, and proposed that benchtop NMR-based metabolomics could be used to monitor type 1 and type 2 diabetes, glucose intolerance, and gestational diabetes, albeit with lower sensitivity than HR-NMR.

Finally, the same group used saliva samples with the same goal of researching biomarkers for type 2 diabetes [83]. This study compared and validated the results using a 60 MHz Magritek Spinsolve Ultra Benchtop spectrometer and a Bruker 400 MHz Avance-1 NMR spectrometer (Coventry, UK). Saliva samples (*n* = 12) were collected from non-smokers (*n* = 6) and smokers of more than 20 cigarettes per day (*n* = 6). The results identified 48 resonances with the 400 MHz spectrometer, 31 of which were endogenous and 4 were exogenous biomolecules. However, only 19 potential biomarkers were detected with the benchtop 60 MHz system, of which 5 showed upregulation and 4 downregulation of metabolites (Table 2). This study had several limitations, such as the low values of resonances becoming similar to coupling constants, which distorted the classic ^1^H multiplet resonance patterns and made it difficult to observe multi-analyte samples. Additionally, smokers had high levels of methanol signal compared to non-smokers, who had little or none of this agent. This xenobiotic signal was inhaled through cigarette smoking and was caused by the combustion of tobacco lignin. However, this signal could have been avoided by refraining from smoking before collecting this biofluid. The same happened with ethanol after consuming alcohol, which were factors that should have been considered during the experimental design. They concluded that the 60MHz benchtop NMR had limitations such as the inability to detect some low concentrate biomolecules in saliva such as guanidoacetate, lactate, and succinate. Future research will need to analyze more potential metabolites that can serve as biomarkers using other types of samples.

### 3.3. Application in Other Conditions

It is expected that the application of benchtop NMR-based metabolomics can be expanded to other clinical situations. As an example, Finch et al. [84] performed a pilot study to evaluate the use of benchtop NMR technology for metabolic profiling of urine samples in cats with chronic kidney disease (CKD). This study was limited by the number of subjects: two healthy controls and two cats with CKD stage 2. However, they were able to identify 15 metabolites in cats with CKD that were different from the controls, including acetate, creatinine, citrate, taurine, glycine, serine, and threonine. These findings suggest that benchtop NMR technology could be a useful tool for supporting clinical decision-making in cats with CKD and potentially other metabolic conditions in the future. Thus, the next step should be to validate this technology in conditions where HR-NMR has been proven to be effective. Early detection, rapid diagnosis, and risk stratification using molecular biomarkers are key strategies for managing noncommunicable diseases such as cancer, respiratory diseases, or renal diseases. Benchtop NMR-based metabolomics has the potential to be used as an affordable screening tool for these conditions.

The first application should be cancer detection. Cancer is the second-leading cause of death globally, accounting for nearly 20% of deaths, according to the World Health Organization. The most frequently diagnosed types of cancer in 2020 were breast (first position; 2,261,419 cases—11.7%), lung (second position; 2206,771 cases—11.4), and colorectal cancer (third position; 1,931,590 cases—10%) (SEOM data https://seom.org/, accessed on 25 November 2022). While there are several effective cancer screening tests available, such as mammography for breast cancer, colonoscopy for colorectal cancer, and low-dose computed tomography for lung cancer, some of these tests are invasive, expose patients to small amounts of radiation, have low sensitivity, or are not widely accessible. Benchtop NMR-based metabolomics has the potential to provide a low-cost alternative for cancer screening. Altered metabolism is a well-known hallmark of cancer, which can be caused by changes in signaling pathways, protein expression, and other molecular mechanisms, and can also reflect specific biochemical adaptations during carcinogenesis that may give malignant cells a survival advantage. This metabolic shift can already be detected in diagnosis through techniques such as the in vivo assessment of glucose metabolism using FDG-PET imaging. NMR-based metabolomics has shown promising results in breast cancer [85], in particular for prognostic and stratification purposes. NMR-based metabolomics has been also used to identify metabolic signatures in urine that can aid in the diagnosis of lung and colorectal cancer. Carrola et al. [86] identified a lung cancer-related NMR-based metabolic signature with an overall classification rate of 93.5%. Brezmes et al. conducted a recent review about metabolites in the urine of colorectal cancers detected by NMR [87]. From the 10 studies analyzed, creatinine, 4-hydroxybenzoic acid, acetone, carnitine, d-glucose, hippuric acid, l-lysine, l-threonine, and pyruvic acid, acetic acid, phenylacetylglutamine, and urea were found to be significantly altered in urine. NMR-based metabolomics has also been explored as a potential diagnostic tool for other types of cancer. The changes in the levels of citrate, choline, and sarcosine aid in the diagnosis of prostate cancer, when compared with controls and benign prostatic hyperplasia [88]. Michálkováet al. [89] investigated the changes of metabolites in plasma samples via NMR and developed a model comprising 12 metabolites (3-hydroxybutyrate, lactate, glutamine, alanine, valine, lysine, citrate, histidine, isoleucine, glutamate, acetone, and dimethylamine) which had an accuracy of 94%, 100% sensitivity, and 90% specificity in distinguishing pancreatic cancer patients from healthy individuals.

The potential of benchtop NMR-based metabolomics is also expected to be useful for early diagnosis of other conditions such as chronic kidney disease [90,91], which has a prevalence of 10–15% in adults worldwide; chronic obstructive pulmonary disease (COPD) [92,93], with an overall incidence rate of 9/1000 PY; or obesity [94,95]. Furthermore, benchtop NMR spectrometers are not limited to metabolomic analysis. They can also be used to monitor specific biomarkers for certain diseases. For example, Dalla Via et al. [96] studied the gut microbiota and the possibility that a compound produced by some bacteria promotes the development of atherosclerosis. Several bacteria taxa (Enterobacteriaceae) had a cutC gene that could convert choline to trimethylamine, which later becomes proatherogenic trimethylamine-N-oxide, which promotes atherosclerosis. Stool samples were collected weekly over three consecutive weeks from 16 healthy adults (four female and 12 male). This gene was analyzed by qPCR, while the presence of choline was examined using a 60 MHz benchtop NMR spectrometer, the Spinsolve 60 Carbon Ultra from Magritek GmbH (Aachen, Germany). They concluded that patients with bacteria containing the cutC gene may metabolize choline and promote the development of atherosclerosis.

## 4. Clinical Sample Analysis: Spectral Analysis Automatization

The use of benchtop NMR for clinical metabolomics analysis requires the ability to process a large number of samples, which in turn necessitates automation. By automating the processing of NMR spectra, we can reduce the dependence on expert operators, ensure consistency in data analysis, and improve the reproducibility of results. In clinical settings, this can be particularly valuable when analyzing large cohorts of patients or when conducting longitudinal studies. Furthermore, automating the analysis of NMR spectra can accelerate the identification and quantification of metabolites, enabling faster and more comprehensive analyses. Ultimately, the goal of using benchtop NMR for clinical metabolomics analysis is to provide a powerful tool for biomarker discovery and personalized medicine, and automation is a key step towards achieving this goal. In this context, developing new software tools and machine learning algorithms can enable more efficient and accurate processing of NMR spectra, ultimately leading to more meaningful insights into disease processes and potential therapeutic interventions.

Automated tools for performing HR-NMR spectral processing analysis already exist [97,98,99,100], allowing for baseline and phase correction, normalization, and signal alignment, all of which are necessary before metabolite quantification and statistical analysis can be performed. While many of these developments have been made for HR-NMR, they can easily be adapted to benchtop NMR systems. To avoid the need for manual spectral fitting and metabolites identification by an expert, software tools have been developed that can automatically perform these steps, reducing the time required to identify and quantify metabolites and improving reproducibility. These software tools mainly use statistical-based algorithms for automatic quantification and can perform tasks such as phase and baseline correction, alignment, binning, and automatic quantification. Several programs have been developed, including BQuant [101], AQuA [102], BATMAN [103], BAYESIL [104], ASICS [105], AlpsNMR [105], and Dolphin/rDolphin [106,107], which can handle both 1D 1H spectra and 2D spectra. These programs are mainly developed for high field NMR and can automate the spectral processing steps.

An alternative approach to spectral processing is the utilization of deep learning algorithms in NMR analysis [108,109,110,111]. Among various deep learning techniques, data pre-processing using convolutional neural networks has demonstrated significant advantages. Moreover, using artificial neural network/deep learning for compound/structure identification and quantification has demonstrated improved performance compared to traditional machine learning methods [108].

In conclusion, the automation of spectral processing steps in NMR-based metabolomic studies is crucial for clinical sample analysis. These automated tools can reduce the time required for spectral processing, improve reproducibility, and avoid the need for manual spectral fitting by an expert. The use of deep learning algorithms has further improved the accuracy and efficiency of metabolite identification and quantification.

## 5. Discussion

Benchtop NMR spectrometers offer several advantages as clinical tools, particularly in terms of sensitivity, versatility, and cost-effectiveness. The limit of detection (LOD) of benchtop NMR can vary depending on the instrument model, sample type, and specific experiment being conducted. However, it is surprisingly and disproportionately high: LOD values of <50 µmol/L for selected metabolites with prominent -CH3 and/or—CH2- function singlet resonances present in aqueous model solutions. SNR values >10 can be achievable at concentrations of ≥25 µmol/L for acetone [112]. Additionally, benchtop NMR can detect a wide range of metabolites, allowing for a more comprehensive analysis of biological samples. The number of metabolites that can be detected by benchtop NMR varies depending on the sample type, instrument model and analysis technique used. For example, Percival et al. identified 20 metabolites in urine using a 60 Mhz magritek Spinsolve NMR spectrometer [21]. Benchtop NMR also has the advantage of being a non-destructive technique, which means samples can be stored and re-analyzed, if necessary. In contrast, many point-of-care approaches require a single-use cartridge, making it impossible to re-analyze samples.

When comparing HR-NMR metabolomic results with benchtop NMR metabolomic results, it is clear that HR-NMR has a higher sensitivity, enabling the detection of metabolites in lower concentrations. This makes HR-NMR particularly useful for analyzing complex mixtures where metabolites may be present in very small amounts. However, benchtop NMR can still detect metabolites at concentrations relevant to most metabolomic studies. In addition, benchtop NMR offers advantages in terms of signal-to-noise ratio and baseline stability, which can improve the accuracy and reproducibility of the data. Therefore, while HR-NMR has a clear advantage in sensitivity, benchtop NMR can still provide reliable and informative data for many metabolomic applications, including clinical diagnosis. In this context, benchtop NMR technology is proving to be effective in detecting developing diseases at an early stage through the use of metabolite fingerprinting at 60 and 80 MHz.

Further validation of this technology in different conditions could have a significant impact on its implementation as an affordable diagnostic tool for the general population. The development of this technology also has the potential to reduce production costs and make it more accessible for use in less developed countries. A main challenge in the development of benchtop NMR spectrometers is creating a uniform magnetic field with compact magnets, but researchers are working on ways to improve passive and active shimming techniques to address this issue. The use of benchtop NMR spectrometers for the analysis of metabolites in body fluids like urine or blood is expected to have a significant impact on healthcare, particularly in the field of personalized medicine. By allowing for the detection of early signs of disease through the analysis of these metabolites, these compact spectrometers could play a key role in the early diagnosis of disease, and the development of more targeted and effective treatments.

## Figures and Tables

**Table 1:** Commercially available benchtop spectrometers. Sensitivity was measured in all cases using 1% Ethylbenzene. Bruker, Magritek, Nanalysis and Oxford Instruments use standard 5-mm NMR tubes, ThermoFisher uses a 0.4-mm capillary and Q Magnetics uses a 1-mm capillary.

**Instrument**	**Nuclei**	**MHz (^1^H)**	**Line Width 50% (Hz)**	**Sensitivity**	**Weight (kg)**	**Lock**	**Dimensions (cm)**	**Autosampler**	**Solvent Suppression**
Bruker Fourier 80 [8]	^1^H, ^1^H/^13^C, ^1^H/^31^P or ^1^H/^129^Xe ^1^H/^13^C|^19^F	80	Standard: 0.4 HD option: 0.3	^1^H-only systems: ≥240 or ≥220 when pulsed field gradient incl. ^1^H/X systems: ≥180 or ≥160 when pulsed field gradient incl. ^1^H/^13^C|19F: ≥120, optimized for X performance	94	External	50 × 70 × 60	Yes	Yes
Magritek Spinsolve 60 [9]	^1^H and ^19^F on all systems + X nuclei for dual channel systems	60	Spinsolve 60: <0.5 Spinsolve 60 Plus: <0.35 Spinsolve 60 Ultra: <0.2	200 (Single channel)/130 (Double channel)	60	External	58 × 43 × 40	Yes	Yes
Magritek Spinsolve 80 [10]	^1^H and ^19^F on all systems + X nuclei for dual channel systems	80	Spinsolve 80: <0.4 Spinsolve 80 Ultra: <0.25	280 (Single channel) 200 (Dual channel)	72.5	External	58 × 43 × 40	Yes	Yes
Magritek Spinsolve 90 [11]	^1^H and ^19^F on all systems + X nuclei for dual channel systems	90	<0.4	>240 (Dual channel)	115	External	66 × 45 × 43	Yes	Yes
Nanalysis 60 MHz [12]	^1^H (60e) ^1^H/^13^C, ^1^H/^31^P, ^1^H/^19^F (60PRO)	60	<1	100	26.3	Internal	30 × 28 × 49	No	Yes
Nanalysis 100 MHz [13]	^1^H (100e) ^1^H/^13^C, ^1^H/^31^P, ^1^H/^19^F (100PRO)	100	<1	220	97	Internal	37.1 × 41.4 × 65.4	No	Yes
Oxford Instruments X-Pulse [14]	^1^H ^19^F ^13^C ^31^P ^11^B ^7^Li ^23^Na ^29^Si	60	<0.35	130	172	Internal	38.5 × 54 × 42.5	Yes	Yes
ThermoFisher picoSpin 80 [15]	^1^H	82	<1.6	>4000	19	Internal	43 × 36 × 25	No	
ThermoFisher picoSpin 45 [16]	^1^H	45	<1.8	>1000	5	Internal	18 × 15 × 29	No	
Q Magnetics QM-125 [17]	^1^H	125	0.55	16	28		37 × 34 × 30	No	No

**Table 2:** Metabolites studies of type 2 diabetes using Benchtop NMR spectroscopy.

**Author/Year**	**Potential Metabolites (n)**	**Upregulated Metabolites**	**Downregulated Metabolites**
Percival et al., 2018 [21]	15	Acetate Alanine Citrate Cn/Creatine Glucose Methylsuccinate 3-D hydroxybutyrate	Aromatic compounds (indoxyl sulphate, hippurate) Formate
Leenders et al., 2020 [82]	5–6	Glucose	Cn/creatine Citrate Hippurate Indoxyl sulfate
Edgar et al., 2021 [79]	15	Citrate Cn/creatine N-acetylated metabolites	Hippurate Indoxyl sulphate 3-(3-hydroxyphenyl)-3-hydroxypropanoate Lactate
Edgar et al., 2022 [83]	19	Acetate Formate Glycine Methanol Propionate	Cn/Creatine Dimethylamine Lysine Trimethylamine
